# Exploring Genomic Variations and Phenotypic Traits of *Chrysodeixis includens* Nucleopolyhedrovirus Isolates to Improve Soybean Pest Control

**DOI:** 10.3390/v17111503

**Published:** 2025-11-14

**Authors:** Lucas A. Andrade, Daniel R. Sosa-Gómez, William Sihler, Bergmann M. Ribeiro, Marlinda L. Souza, Rogerio B. Lopes, Daniel M. P. Ardisson-Araújo

**Affiliations:** 1 Laboratory of Insect Virology and Phages, Cell Biology Department, University of Brasilia, Brasilia 70910-900, DF, Brazil; lucas-andrade.la@aluno.unb.br; 2 Department of Entomology, Embrapa Soybean, CP 4006, Acesso Orlando Amaral, Londrina 86085-981, PR, Brazil; daniel.sosa-gomez@embrapa.br; 3 Embrapa Genetic Resources and Biotechnology, Biological Station Park, Brasília 70770-917, DF, Brazil; william.sihler@embrapa.br (W.S.); marlinda.souza@embrapa.br (M.L.S.); rogerio.lopes@embrapa.br (R.B.L.); 4 Laboratory of Baculovirus, Cell Biology Department, University of Brasilia, Brasilia 70910-900, DF, Brazil; bergmann@unb.br

**Keywords:** Plusiinae, *Alphabaculovirus chrincludentis*, biological control, pathogenicity, comparative genomics, photoprotectant

## Abstract

Soybean production is a cornerstone of Brazilian agriculture but is heavily threatened by insect pests such as the soybean looper *Chrysodeixis includens*, capable of reducing yields by up to 70% if uncontrolled. Reliance on chemical insecticides is increasingly unsustainable due to environmental impacts and resistance, highlighting the need for eco-friendly alternatives. The alphabaculovirus *Chrysodeixis includens* nucleopolyhedrovirus (ChinNPV) is an important biocontrol agent largely used in Brazilian fields because of its host specificity and safety, although its persistence is limited by ultraviolet (UV) sensitivity. Here, we characterize two ChinNPV isolates, CNPSo-168 (C168) and Tabatinga (Tb), using genomic and phenotypic analyses. Whole-genome sequencing revealed circular dsDNA genomes of 139,290 bp (154 ORFs) for C168 and 139,131 bp (153 ORFs) for Tb, both encoding the 38 baculovirus core genes and sharing >98.9% identity with reference genomes. Comparative genomics identified 431 SNPs, including 132 nonsynonymous changes in structural, regulatory, and infection-related genes. At low concentrations, C168 showed an approximately 2-fold lower LC_50_ than Tb (higher potency), while both achieved near-complete mortality within 8 days at higher concentrations. This greater potency at lower concentrations reinforces the efficacy-based rationale for selecting isolate C168 for biocontrol applications. Infection reduced larval growth, pupation, and adult emergence, often with developmental impairments. Despite genetic differences, both isolates were highly UV-sensitive, and formulation tests indicated that titanium dioxide combined with kaolin conferred partial protection. These results provide insights into ChinNPV diversity and support its development as a sustainable tool for soybean pest management.

## 1. Introduction

Herbivorous insect pests cause an estimated reduction in global agricultural productivity of 20–40% [1]. In Brazil, soybean pests are responsible for an average annual yield loss of 7.7% [2]. Among these pests, the soybean looper *Chrysodeixis includens* (Lepidoptera: Noctuidae) is particularly damaging, with high yield reductions by defoliation reported since the 2000s [3]. Although chemical insecticides remain widely used, their long-term efficacy is undermined by environmental contamination, negative effects on non-target organisms, and the rapid evolution of resistance, underscoring the urgent need for sustainable alternatives [4]. Baculovirus-based biopesticides are increasingly used in Brazil primarily because of their proven efficacy under field conditions, with host specificity and safety to non-target organisms as additional advantages [4].

*Chrysodeixis includens* nucleopolyhedrovirus (ChinNPV), a member of the genus *Alphabaculovirus* (family *Baculoviridae*), is a large dsDNA. It produces occlusion bodies (OBs), a protective matrix composed primarily of the viral protein polyhedrin [5]. These OBs are the active ingredient in agricultural biocontrol. ChinNPV is a promising agent because of its efficacy, killing larvae within 5–10 days and reducing feeding damage by up to 95% [6,7]. Following efficacy as the main driver of use, its high host specificity preserves beneficial insects [8], and its distinct mode of action mitigates the risk of cross-resistance with chemical insecticides [9]. Field studies confirms that commercial ChinNPV formulations are as effective as chemical controls and are compatible with standard herbicides and fungicides, facilitating the integration into integrated pest management (IPM) programs [7,9].

Despite these advantages, the protection conferred by OBs against desiccation and heat does not extend to ultraviolet (UV) radiation, which induces DNA damage (e.g., pyrimidine dimers and strand breaks) or generates reactive radicals that inactivate virions [10,11,12]. Consequently, UV sensitivity remains a major limitation to the field application of baculoviruses [13]. Variation in UV tolerance has been reported both among baculovirus species and among isolates of the same species, indicating a genetic basis for UV stability [14,15,16]. Both experimental selection and genetic engineering, for instance, the incorporation of cyclobutane pyrimidine dimer-*photolyase* (*cpd*-*phr*) genes, have successfully enhanced UV resistance [17,18,19,20,21]. Understanding the UV response of ChinNPV, which naturally carries a *cpd-phr*, is therefore critical for assessing persistence under field conditions and optimizing application in integrated pest management (IPM) programs [17,18,19,20,21].

ChinNPV represents a promising alternative to chemical insecticides in soybean pest management [22]. However, fully realizing this potential requires characterizing the genetic diversity and biological activity of ChinNPV isolates to identify traits that enhance performance in IPM, especially against pesticide-resistant *C. includens* populations [8]. The analysis of ChinNPV populations has revealed a remarkable degree of natural genetic variability, consistent with findings in other baculoviruses. For example, Craveiro et al. [23] identified five distinct isolates from just seven infected larvae collected at different times and locations in Brazil, highlighting the geographical structuring of ChinNPV populations. Similarly, plaque purification of field material has shown that even small sample sets can harbor substantial diversity, with up to 23 genotypic variants identified from only 11 larvae in a soybean field [24]. This level of variability surpasses that observed in the closely related Chrysodeixis chalcites NPV (ChchNPV), in which only four isolates were identified from 103 infected larvae out of more than 4000 collected insects in the Canary Islands [25]. Because the biological performance of baculoviruses can vary across environmental contexts and host populations, characterizing indigenous isolates from different geographical regions is essential to identify those best adapted for local field deployment and sustainable pest management.

In this study, we investigated two novel ChinNPV isolates, CNPSo-168 (C168) and Tabatinga (Tb), to link genomic variations with biological performance. These isolates were selected because a pilot bioassay in our laboratory indicated that C168 caused higher mortality at lower concentrations than Tb, suggesting biologically meaningful phenotypic variation between them. We evaluated infection outcomes under sublethal exposure, pupation and adult emergence effects, and viral stability under UV radiation with and without photoprotective agent. Whole-genome sequencing revealed genomic variations relative to reference strains, including differences in open reading frame (ORF) content, while phylogenetic analysis confirmed placement within the ChinNPV clade. By integrating phenotypic and genomic data, this study highlights subtle but relevant differences that may influence pathogenicity, speed of kill, transmissibility, and persistence, supporting more effective biocontrol strategies for sustainable soybean production.

## 2. Materials and Methods

### 2.1. Insect Rearing, Virus Amplification, Purification and Quantification

Two viral isolates were used in this study: ChinNPV-CNPSo-168 (BRM 064873; designated ChinNPV-C168) and ChinNPV-Tabatinga (BRM 063868; designated ChinNPV-Tb). C168 was originally isolated from infected *C. includens* larvae collected in soybean fields in Londrina (Paraná State), whereas Tb was obtained from larvae collected in the agricultural colony of Tabatinga, Planaltina (Federal District). Viral amplification was performed using third- and fourth-instar *C. includens* larvae obtained from two independent insect colonies, sourced from Pragas.com (Piracicaba, SP, Brazil) and EMBRAPA Genetic Resources and Biotechnology (Brasília, DF, Brazil). For each amplification replicate, larvae (*n* = 120, including 15 unexposed control larvae) were individually placed in 30 mL plastic cups containing a formol-free artificial diet. The diet was prepared by mixing white bean flour (125 g), brewer’s yeast (62.4 g), wheat germ (100 g), agar (63 g), soy protein (100 g), powdered milk (150 g), and distilled water (1900 mL). The mixture was autoclaved at 121 °C for 20 min and cooled to approximately 50 °C before adding the following heat-sensitive components: ascorbic acid (6000 mg), sorbic acid (3000 mg), methyl paraben (5000 mg), formaldehyde 40% (6 mL), hydrochloric acid (10 mL), and a vitamin solution (10 mL). The vitamin solution consisted of niacinamide (1000 mg), calcium pantothenate (1000 mg), riboflavin (500 mg), thiamine (250 mg), pyridoxine (250 mg), folic acid (100 mg), biotin (20 mg), vitamin B_12_ (2 mL of a 1000 mg/mL stock solution), and distilled water up to 1000 mL. After homogenization, the diet was dispensed into sterile trays, allowed to solidify at room temperature, and stored at 4 °C until use.

The diet surface was treated 100 µL with each viral suspension at a concentration of 1.00 × 10^7^ OBs/mL [23]. Larvae were allowed to feed up to 10 days or until 100% mortality was observed. Cadavers were collected and homogenized in a buffer (1% ascorbic acid, 2% SDS, 0.01 M Tris pH 7.8, 0.001 M EDTA) using a ratio of 0.5 mL of buffer per 1 g of larval tissue. The homogenate was filtered through three layers of gauze, and the filtrate was centrifuged at 7000× *g* for 15 min. The resulting pellet was resuspended in TE buffer (0.01 M Tris pH 7.8, 0.001 M EDTA) and centrifuged again at 10,000× *g* for 12 min. The final pellet was resuspended in TE buffer, and the OB concentration was quantified using a Neubauer hemocytometer at a 1:1000 dilution [24]. Purified OB stocks were stored at −20 °C. Importantly, SDS was not applied directly to purified OBs but used only during the initial homogenization of larval tissues to facilitate carcass disruption; therefore, the effective SDS exposure to OBs was markedly reduced by dilution and subsequent washing steps. Since both ChinNPV isolates (C168 and Tb) were processed identically, this step does not affect comparative analyses.

### 2.2. Viral DNA Extraction and Quantification

Viral DNA was extracted using a phenol/chloroform/isoamyl alcohol solution (25:24:1). First, 125 μL of alkaline buffer (0.3 M Na_2_CO_3_, 0.5 M NaCl, 0.03 M EDTA) was added to 250 μL of viral suspension (1 × 10^9^ OBs/mL) and incubated at 37 °C for 30 min. Then, 12.5 μL of 20% SDS was added and incubated for 10 min, followed by 12.5 μL of proteinase K (20 mg/mL) and incubation for 6 h or overnight at 37 °C. Next, the samples were centrifuged three times at 16,000× *g* for 3 min and sequentially treated with 400 μL phenol, 400 μL phenol/chloroform/isoamyl alcohol (25:24:1), and 400 μL chloroform/isoamyl alcohol (24:1), transferring the aqueous phase each time to a clean tube. DNA was then precipitated with 500 μL of cold 70% ethanol, centrifuged for 10 min, washed with 1 mL of 100% ethanol, and centrifuged again for 30 min. After drying the pellet, genomic DNA was resuspended in 50 μL of Milli-Q ddH_2_O. DNA concentration was measured using a NanoDrop spectrophotometer (Life Plus, Waltham, MA, USA). DNA quality was checked by 0.8% agarose gel electrophoresis, stained with 0.01% ethidium bromide, and visualized under UV light using an AlphaImager Mini system (Alpha Innotech, San Leandro, CA, USA).

### 2.3. Genome Sequencing, SNP Detection and Phylogenetic Analysis

Genomic DNAs from the ChinNPV-C168 and ChinNPV-Tb isolates was sequenced on an Illumina HiSeq 2000™ platform (paired-end reads) by ZymoResearch (Irvine, CA, USA). For each isolate, approximately 5 μg of purified DNA was submitted. Raw reads were quality-filtered and assembled in Geneious R19 [26] using the ChinNPV-IE genome (KJ631622) as a reference. Open reading frames (ORFs) were predicted in Geneious using the following criteria: initiation codon (ATG), a minimum product size of 50 amino acids, and minimal overlap with adjacent ORFs. Predicted ORFs were validated using BLASTx v. 2.15.0 searches against the NCBI non-redundant protein database [27]. The consensual genomes of ChinNPV-C168 and Tb were deposited in the Genbank under the accession numbers PX425276 and PX425275, respectively.

For comparative analysis, the assembled genomes were aligned with those of 14 other ChinNPV isolates. Nucleotide identity across homologous genes was assessed using pairwise BLASTx alignments [27]. A phylogenetic three was reconstructed form a whole genome alignment generated with MAFFT v7.308 [28]. The alignment was used to infer a midpoint-rooted tree under a General Time Revesible (GTR) model in FastTree [29]. Branch support was evaluated using the Shimodaira–Hasegawa-like approximate likelihood ratio test (maximum support value = 1). Finally, overall, pairwise nucleotide identity between isolate genomes was calculated directly from the MAFFT alignments.

### 2.4. Pathogenicity Bioassays: Lethal Concentration and Sublethal Effects

Lethal concentration (LC) assays were conducted for both ChinNPV-C168 and ChinNPV-Tb isolates. Viral suspensions were prepared at the following concentrations: 1.00 × 10^4^, 5.00 × 10^4^, 1.00 × 10^5^, 5.00 × 10^5^, 1.00 × 10^6^, 5.00 × 10^6^, and 1.00 × 10^7^ OBs/mL. A water-only treatment served as the negative control. For each viral concentration and the control, recently molted 3rd-instar *C. includens* larvae (12–18 h after ecdysis) were starved for 14 h and then individually placed in 24-well plates. Each larva was provided with a standardized solid diet disk prepared by dispensing liquid artificial diet into microtubes, allowing it to solidify, and cutting it into disks of approximately 1.5 cm in diameter and 0.5 cm in thickness (≈1.0–1.2 g). The disks were visually inspected and weighed to ensure uniformity across treatments. The surface of each disk was treated with 30 μL of the corresponding OB suspension or water. All larvae used in the bioassays originated from the same synchronized colony batch to ensure uniform developmental stage and physiological condition. After a 24 h exposure period, all surviving larvae were transferred to individual 30 mL plastic cups containing virus-free artificial diet, which was replaced every three days. Mortality was recorded daily for 10 days. All concentrations were tested to characterize the full mortality response range; however, only doses ranging from 5.00 × 10^5^ to 1.00 × 10^7^ OBs/mL were used to calculate LC_50_ and LC_95_ values, as the lowest two concentrations produced minimal mortality and affected the model fit. LC_50_ and LC_95_ values were calculated from three independent replicates per concentration (*n* = 30 larvae per replicate, total = 90 per concentration). For the time-to-death assays, the same infection protocol was applied using a single viral challenge concentration equivalent to the calculated LC_95_ value for each isolate. The two lowest concentrations (1.00 × 10^4^ and 5.00 × 10^4^ OBs/mL) were also used to evaluate sublethal parameters, including the ability of larvae to reach the pupal stage and the proportion of adults that successfully emerged from pupae. These insects were reared individually on a virus-free diet until pupation. The resulting adults were maintained in 2 L beakers, provided with a liquid diet, and observed until natural death to record longevity and fecundity.

### 2.5. Effect of UV-C Exposure and Formulation with UV Protectants

Time-to-death bioassays were conducted to evaluate differences in susceptibility of ChinNPV isolates (C168 and Tb) to UV-C exposure, with or without UV-protective formulations. Viral suspensions (1.00 × 10^7^ OBs/mL) were sprayed onto soybean (*Glycine max*) leaf disks (4.5 cm of diameter) using a spray tower (~1.50 × 10^4^ OBs/cm^2^), while water-treated disks served as controls. After drying, disks were exposed to UV-C light (245 nm, HNS S 30W T8-OSRAM [OSRAM GmbH, Munich, Germany]) for 2.5 or 5 min (35 cm distance), or kept unexposed. Groups of 20 larvae at 3rd-instar were allowed to feed for 24 h, then transferred to individual wells with artificial diet. Larval mortality was recorded daily for 10 days. For formulation tests, a wettable powder (WP) was produced by mixing C168 OB suspension (8.00 × 10^9^ OBs/mL) with kaolin (1:2 *w*/*w*), dried under controlled conditions (‘water activity’, Aw = 0.04), and gently ground using a glass rod to yield ~4.00 × 10^9^ OBs/g. WP and non-formulated suspensions (4.00 × 10^8^ OBs/mL) were adjusted to 1.00 × 10^7^ OBs/mL, with or without TiO_2_ (10% *w*/*w*) added to the viral suspension before mixing with kaolin as a UV blocker. Treated disks were irradiated or not and assayed as above. All treatments were performed in triplicate with independent viral and insect cohorts.

### 2.6. Statistical Analyses

Mortality data were analyzed using logit regression with a quasi-binomial error distribution in R Statistical Software v. 4.5.0 [30] to estimate LC_50_ and LC_95_ values with 95% confidence intervals. GraphPad Prism v. 8.0.2 (GraphPad Software, Inc. [Boston, MA, USA]) was used for graphical representation. Analyses of sublethal effects and survival were performed using R Statistical Software [30]. Pupae formation and adult emergence rates of survived larvae exposed to different viral concentrations were fitted to generalized linear models (GLM) with binomial distribution (logit-link function). A GLM with Poisson distribution was used for analysis of the larval growth. The goodness-of-fit of these models were verified for each dataset using the ‘hnp’ package. Multiple pairwise comparisons between treatments were performed with estimated marginal means using the ‘emmeans’ package (*p* < 0.05). The Kaplan–Meier method (package survival) was employed to estimate survival curves and median survival times (ST_50_). Survival curves were compared using the χ^2^ log-rank test, with pairwise comparisons adjusted using the Bonferroni correction (*p* < 0.05). ST50 values were calculated from the fitted Kaplan–Meier survival curves using the ‘survfit’ function, which allows interpolation between 24 h observation intervals; therefore, decimal ST50 values reflect estimated survival probabilities rather than exact observation times. All raw data and additional information supporting the findings of this study are available from the corresponding author upon reasonable request.

## 3. Results

### 3.1. Genome Sequencing and Description, and Comparative Genomics of ChinNPV Isolates

After trimming low-quality reads and performing pairwise alignments, isolate C168 yielded 2,453,865 reads (156.6 ± 66.6 nt), whereas Tb produced 782,587 reads (165.5 ± 63.1 nt). Genome assembly was conducted using the ChinNPV-IE genome as a reference. The consensual circular genome of C168 measured 139,290 bp with an average coverage of 2764.6× ± 484.9 and a G + C content of 39.2%. The consensual Tb genome was slightly smaller, spanning 139,131 bp with 937.0× ± 180.8 coverage and the same G + C content. We annotated 154 ORFs in C168 and 153 in Tb, covering 91.5% and 91.2% of their respective genome lengths (Tables S1 and S2). Both isolates contained the complete set of 38 core baculovirus genes. In addition, 14 ORFs in C168 and 13 in Tb, although present in previously published ChinNPV genomes, were newly annotated here (Tables S1 and S2). Consistent with Group II alphabaculoviruses, both genomes possess an *f protein* homolog but lack *gp64*.

All predicted ORFs were compared to previously described ChinNPV and other baculovirus sequences using BLASTX. ORF 154 was uniquely annotated in C168 and absent in Tb due to a single point mutation. Several ORFs displayed similarity to sequences from other baculoviruses or even unrelated organisms such as bacteria and ascoviruses, although all were also present in other ChinNPV isolates (Tables S1 and S2). Apart from this, the ORF content was identical between the two genomes. As summarized in Table 1, the genome sizes of C168 and Tb fall within the established ChinNPV range (138.7–140.8 kb). Notably, C168 harbors the highest number of annotated ORFs (154) among all isolates analyzed to date, followed by Tb (153), whereas previously reported genomes typically contained 140–142 ORFs. This highlights the more comprehensive annotation achieved in this study. No homologous regions were identified in either genome, consistent with observations for other ChinNPV isolates. Furthermore, whole-genome alignments revealed strict gene collinearity and complete conservation of genome architecture across all ChinNPV isolates, including C168 and Tb, with no evidence of rearrangements, inversions, or structural variation, indicating highly conserved synteny within this species.

### 3.2. Pairwise Nucleotide Identity and Phylogenetic Analysis of ChinNPV Isolates

**Figure 1:**
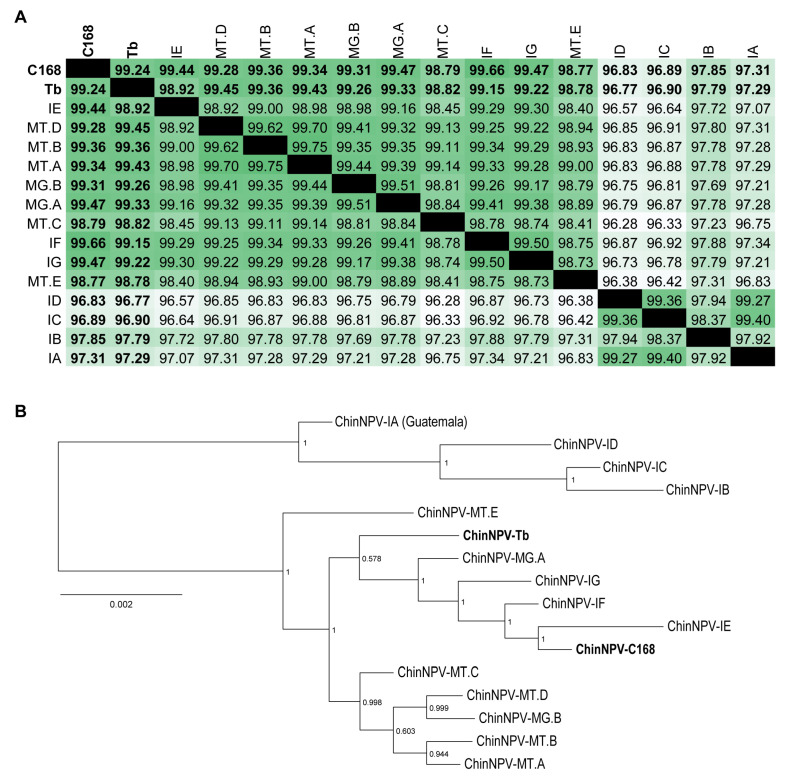
Comparative genomic analysis and phylogenetic relationships among the two novel ChinNPV isolates. (**A**) Heatmap of pairwise nucleotide identity across complete genomes aligned with MAFFT. Darker shades of green indicate higher identity. (**B**) Maximum Likelihood phylogenetic tree inferred from full-genome alignments of ChinNPV isolates using MAFFT and FastTree with GTR evolutionary model. Branch support values were calculated using a Shimodaira–Hasegawa-like test (maximum = 1). MG = Minas Gerais; MT = Mato Grosso. The two isolates characterized in this study are highlighted in bold, CNPSo-168 (C168) and Tabatinga (Tb).

Pairwise nucleotide identity analysis based on full-genome alignment using the MAFFT revealed that C168 showed the highest identity (99.4%) with ChinNPV-IE, indicating it as the closest relative (Figure 1A). Importantly, ChinNPV-IE (the genome used to assembly the novel isolates) was collected in the city of Iguaraçu, Paraná State (Brazil), in January 2007, while the C168 isolate was collected in the same city in December 2013, suggesting a common ancestry of circulant virus population, both as natural epizootic infections. On the other hand, the Tb isolate was most similar to ChinNPV-MG.A (99.33% identity), which was collected in Buritis, Minas Gerais State in February 2014. Also, Tb was found to be closely related to isolates ChinNPV-IG, -IF, -IE, and C168 (Figure 1A). The Tb isolate was collected in a rural area near the city of Planaltina, Federal District (Brazil), which is geographically closer to the city of Buritis, Minas Gerais, where the isolates -MG.A and -MG.B were found.

A phylogenetic tree was generated using the FastTree method based on the whole-genome nucleotide alignment of the two novel ChinNPV isolates (C168 and Tb) alongside 14 previously published ChinNPV genomes available in GenBank (Table 1). Both C168 and Tb clustered within the same branch (Figure 1B). On the other hand, Tb was positioned closer to the most recent common ancestor (m.r.c.a.) of the clade, suggesting it diverged earlier or followed a distinct evolutionary pattern compared to C168 (Figure 1B). Another isolate, ID, was also collected in Iguaraçu but clustered more closely with the clade formed by the isolates IB, IC, and IA, which were collected in Londrina (January 2006), Maringá (January 2006), and Guatemala (1972), respectively. Interestingly, the sister clade to the one containing Tb and C168 included isolates from both Mato Grosso (MT.A, MT.B, MT.C, and MT.D) and Minas Gerais (MG.B). Although some clustering by geographic origin was observed among ChinNPV isolates (Figure 1B), a formal test of association between geographic distance and phylogenetic divergence was not performed due to the limited number of isolates with precise collection metadata. Therefore, no inference of spatial genetic structure was drawn from these data.

The strong branch support values validated the close relationship between the two novel isolates, as both C168 and Tb clustered within the same branch of the phylogenetic tree (Figure 1B). The overall topology confirmed their classification within the species *Alphabaculovirus chrincludentis*. This was further supported by their short branch lengths relative to other ChinNPV isolates (Figure 1B). Moreover, pairwise nucleotide distance analyses using the Kimura 2-parameter (K2P) model for the core genes *lef-8*, *lef-9*, and *polh* revealed divergence values well below the 0.072 threshold established for baculovirus species demarcation, thereby confirming the conspecific status of C168 and Tb.

### 3.3. Nucleotide Variations in the ChinNPV Isolates

The whole-genome alignment between the consensual genomes of C168 and Tb isolates revealed genotypic divergence, with 431 single-nucleotide polymorphisms (SNPs) identified across their genomes (Table S3). Using C168 as the reference, we mapped the SNPs present in Tb and found that 299 were synonymous, causing no change in the predicted encoded proteins, while 132 were non-synonymous, resulting in amino acid substitutions in Tb, when compared to C168. Among the affected genes, *bro-a* exhibited the highest level of variability, accumulating 24 SNPs, 14 of which were non-synonymous and one frameshift mutation that disrupted the reading frame in the Tb genome. While most substitutions involved residues with similar physicochemical properties, 14 SNPs caused changes in the side-chain, potentially impacting protein function. Several other genes also harbored significant non-synonymous variation. In *p74*, a gene associated with oral infectivity, five of 14 SNPs resulted in amino acid changes, potentially affecting host entry. The ribonucleotide reductase-coding gene, *rr1* accumulated six non-synonymous SNPs out of 13, which may influence replication dynamics. ORF5 had six non-synonymous changes out of seven SNPs and codes for a hypothetical protein. Other highly polymorphic genes include *hoar*, ORF7, and ORF127 (Table S3). Interestingly, despite the genomic divergence detected between these isolates, no SNPs were found in *phr* gene.

Beyond these, structural and regulatory genes were also affected. The *polyhedrin* gene displayed an R175K substitution, possibly influencing OB formation. Genes involved in replication (*dnapol*, *dbp*, *helicase*), transcription (*lef-4*, *lef-6*, *lef-8*, *lef-11*) and host manipulation (*fgf*, *egt*, *ptp*) also showed non-synonymous SNPs. Structural genes like envelope and nucleocapsid-related genes such as *odv-e66*, *fp25K*, *pif-1*, *pif-3*, and *vp39* were similarly affected.

### 3.4. Lethal Concentration for ChinNPV Isolates and Biological Parameters in Sublethal-Treated Insects

**Figure 2:**
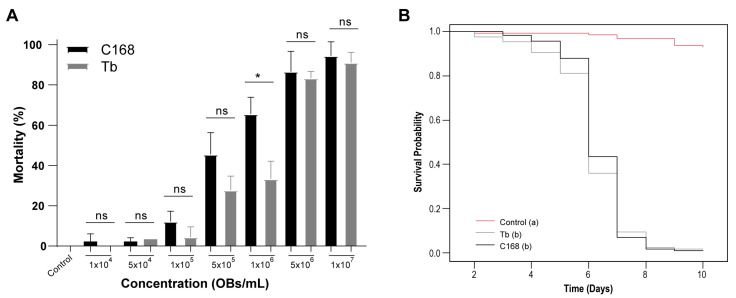
Concentration-response and survival of ChinNPV isolates CNPSo-168 (C168) and Tabatinga (Tb) in 2nd- to 3rd-instar *C. includens* larvae. (**A**) Lethal concentration assay. No virus-like symptoms were observed in the negative control, and asymptomatic mortality remained below 2.5%. A significant difference between isolates was detected only at 1.00 × 10^6^ OBs/mL (*t*-test, *p* < 0.001, *). (**B**) Survival curves for larvae infected with C168, Tb, or untreated control. No virus-related mortality was observed in the control group, and asymptomatic deaths did not exceed 2%. Survival differed significantly among treatments (log-rank test, *p* < 0.001). Letters in parentheses indicate significant differences between treatments; ns = not significant.

Given the genomic divergence identified between the two isolates, particularly in genes related to infection and replication, we next assessed whether these differences were reflected in pathogenicity (LC_50_/LC_95_) by performing concentration-mortality bioassays. The two isolates, C168 and Tb, exhibited distinct lethality profiles (Table 2 and Figure 2). Infected larvae from both treatments displayed integument rupture and the characteristic tree-top disease phenotype. Across all tested concentrations, C168 consistently tended to induce higher mortality, especially at lower concentrations (Figure 2A). However, a statistically significant difference between the isolates was detected only at 1.00 × 10^6^ OBs/mL.

The estimated LC_50_ for C168 was 5.62 × 10^5^ OBs/mL (95% fiducial limits: 4.16 × 10^5^ to 7.58 × 10^5^), whereas Tb showed a higher LC_50_ of 1.19 × 10^6^ OBs/mL (95% fiducial limits: 9.49 × 10^5^ to 1.49 × 10^6^), indicating that C168 was ~2-fold more potent at lower concentrations (Table 2). At higher concentrations, larval mortality approached but did not reach complete lethality in some replicates, and the LC_95_ values, 8.90 × 10^6^ OBs/mL for C168 (95% fiducial limits: 4.62 × 10^6^ to 1.72 × 10^7^) and 1.62 × 10^7^ OBs/mL for Tb (95% fiducial limits: 9.64 × 10^6^ to 2.73 × 10^7^), were estimated by model extrapolation from the fitted logit regressions. Both isolates exhibited adequate goodness-of-fit (C168: χ^2^ = 21.4, df = 13, *p* = 0.064; Tb: χ^2^ = 14.2, df = 13, *p* = 0.362) and dispersion parameters close to 1 (φ = 1.65 and 1.09, respectively), confirming reliable model performance. Survival analysis revealed significant differences between both virus treatments and the control (log-rank test: χ^2^ = 306, df = 2, *p* < 0.001), but no significant difference between C168 and Tb. The median ST_50_ was six days for both isolates, and by day 8 all virus-exposed larvae had succumbed to infection (Figure 2B; Table 2).

**Figure 3:**
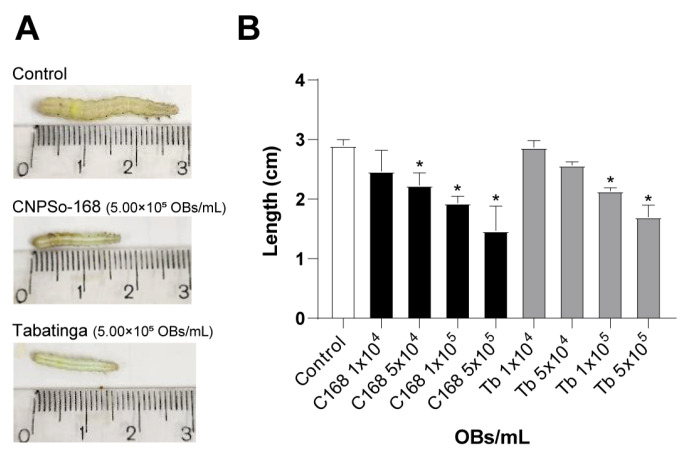
Growth inhibition in *Chrysodeixis includens* larvae treated with ChinNPV isolates CNPSo168 (C168) and Tabatinga (Tb). (**A**) Representative larva from the control group (not exposed to viral suspension) and from groups treated with either C168 or Tb isolates at a concentration of 5.00 × 10^5^ OBs/mL. All larvae were measured 11 days post-inoculation. (**B**) Mean larval length (cm) of *C. includens* following infection with either C168 or Tb. Error bars indicate standard deviation. Asterisks (*) denote statistically significant differences from the control group (GLM with Poisson distribution followed by pairwise comparisons using the emmeans package, *p* < 0.001).

To assess the sublethal effects of ChinNPV infection on host development, we measured larval growth under different viral concentrations. Larvae from the virus-free control group reached an average length of 2.9 ± 0.1 cm (Figure 3A). In contrast, larvae exposed to the C168 and Tb isolates showed concentration-dependent growth inhibition and concentrations higher than 5.00 × 10^4^ OBs/mL for C168 and 1.00 × 10^5^ OBs/mL for Tb differed from untreated larvae (χ^2^ = 2.71; df = 8; *p* < 0.001) (Figure 3B). For C168, average larval length decreased progressively with increasing viral concentration (from 2.47 ± 0.35 cm at 1.00 × 10^4^ OBs/mL to 1.47 ± 0.42 cm at 5.00 × 10^5^ OBs/mL), similarly to that observed for Tb-treated larvae (from 2.87 ± 0.12 cm at 1.00 × 10^4^ OBs/mL to 1.70 ± 0.20 cm at 5.00 × 10^5^ OBs/mL). Notably, C168 induced a tendence of greater reduction in size than Tb at intermediate concentration (5.00 × 10^4^ OBs/mL), indicating a potential stronger impact.

To further evaluate sublethal impacts of ChinNPV infection, we analyzed pupation and adult emergence rates in *C. includens* following exposure to different viral concentrations. Both ChinNPV isolates negatively affected *C. includens* development in a concentration-dependent manner, reducing the percentage of survived larvae that successfully reached pupation and adult emergence compared to the 100% rate observed in the control group (Table 3). For C168, pupation ranged from 86.46% at 1.00 × 10^4^ OBs/mL to 62.85% at 5.00 × 10^5^ OBs/mL, while adult emergence declined from 77.91% to 35.47% over the same range (Table 3). Statistically significant reductions (*p* < 0.001) were detected only at the highest concentration. The Tb isolate caused significant reductions at all tested concentrations, with pupation rates decreasing from 84.98% to 51.40% and adult emergence from 75.09% to 43.06% as the concentration increased. A high incidence of larval mortality during the transition to the pupal stage was observed, with many individuals failing to complete metamorphosis and forming aberrant pupal structures. Some moths from the virus-treated groups displayed wing deformities that would impair flight, likely leading to mortality under natural conditions. Moths from both control and treated groups successfully reproduced and laid eggs (data not shown). We did not test for the presence of the virus; therefore, we cannot confirm its complete clearance.

### 3.5. Effect of UV-C Radiation on ChinNPV Isolates Inactivation in Association with Protective Agents

**Figure 4:**
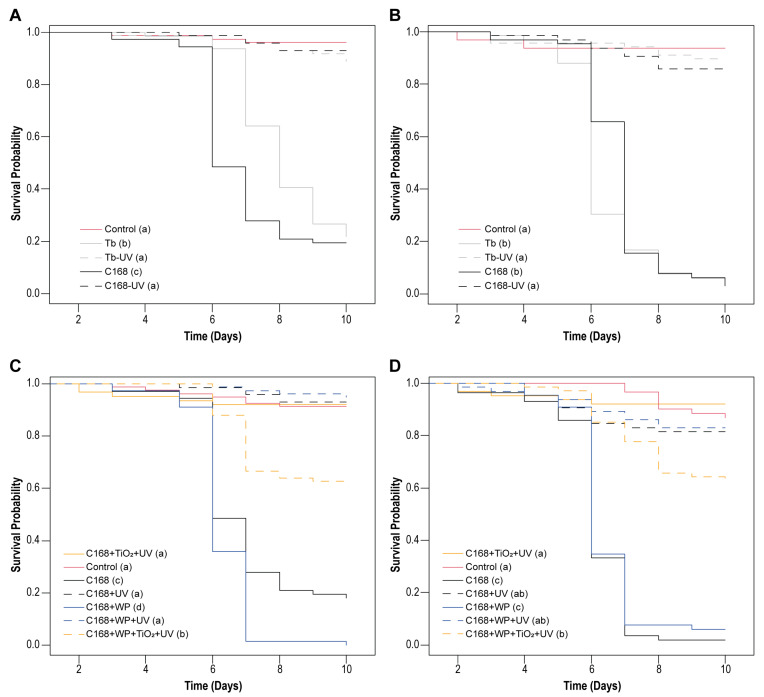
Survival curves for *C. includens* larvae treated with ChinNPV isolates occlusion bodies (OBs) on soybean leaf disks. (**A**,**B**) Isolates CNPSo-168 (C169) and Tabatinga (Tb) isolates exposed or not to UV-C radiation for (**A**) 5 min or (**B**) 2.5 min. (**C**,**D**) C168 isolate associated or not with UV blockers (WP, wettable powder = kaolin and TiO_2_ = titanium dioxide) and exposed or not to UV-C radiation for (**C**) 5 min or (**D**) 2.5 min (**D**). No virus-related mortality was observed in the control group, and asymptomatic deaths did not exceed 12%. Survival curves within all assays differed significantly among treatments (log-rank test, *p* < 0.001). Letters in parentheses indicate significant differences between treatments.

After demonstrating concentration-dependent lethal concentration, effects on infected larval development, and the sublethal effects of both isolates on larval pupation and adult emergence, we evaluated whether UV-C exposure could further influence virus infectivity and the impact on host survival. The survival analysis of *C. includens* larvae confirmed the high sensitivity of ChinNPV OBs to UV-C radiation (Figure 4A,B). Significant differences in survival curves were observed among treatments after exposure of viral OBs to 5 min (χ^2^ = 239; df = 4; *p* < 0.001) and 2.5 min (χ^2^ = 266; df = 4; *p* < 0.001) of UV-C irradiation for both isolates (Figure 4A,B). When larvae were fed on leaf disks treated with the C168 and Tb OBs isolates previously exposed to UV-C, no mortality was recorded, and survival curves closely resembled those of the uninfected control group, confirming the complete inactivation of viral OBs under these conditions. In contrast, non-irradiated viruses retained full activity, with ST_50_ values ranging from 6 to 8 days for both isolates, indicating comparable speed of kill (ST_50_) in the absence of radiation.

Since C168 consistently exhibited higher lethality at lower concentrations, formulation assays were performed exclusively with this isolate. When combined with UV blockers and subsequently exposed to UV-C, C168 retained infectivity, as evidenced by significant reductions in larval survival after both 5 min (χ^2^ = 392; df = 6; *p* < 0.001) and 2.5 min (χ^2^ = 295; df = 6; *p* < 0.001) of irradiation (Figure 4C,D). The kaolin-based wettable powder (WP) formulation did not negatively affect larval feeding or mortality relative to the unformulated virus but offered no protection against UV-C irradiation, with similar ST_50_ values for WP and unformulated (6 days) treatments. By contrast, incorporation of TiO_2_ into the WP formulation partially reduced OB inactivation, increasing larval infection compared to the irradiated, unformulated OBs. Although we were not able to estimate ST_50_ values in this case, survival curves of C169 + TiO_2_ + kaolin were different from unformulated and kaolin-formulated C169 for the longer exposure time (5 min). Larval mortalities reached 37.57% for TiO_2_ + kaolin-based formulation and less than 8% for unformulated and kaolin-formulated after 10 days after UV-C exposure. These results confirm that ChinNPV isolate are inherently UV-sensitive and highlight that while inert carriers such as kaolin are ineffective, TiO_2_-based formulations represent a promising strategy to enhance viral persistence and efficacy under treatments resembling field conditions.

## 4. Discussion

In this study, we sequenced, annotated, and compared two novel isolates of Chrysodeixis includens nucleopolyhedrovirus (ChinNPV), C168 and Tb. In Brazil, field deployment of ChinNPV is primarily driven by efficacy, with host specificity and safety considered important but secondary advantages. Genomic analyses revealed high overall similarity to previously described ChinNPV genomes but also uncovered distinctive polymorphisms, including numerous non-synonymous SNPs in genes associated with infectivity, replication, and structural functions. Biological assays confirmed that both isolates are pathogenic to *C. includens*, inducing concentration-dependent mortality and effects on larval growth and sublethal effects on pupation and adult emergence. Notably, C168 exhibited greater potency (~2-fold lower LC_50_) at lower concentrations, while both isolates were highly sensitive to UV-C inactivation. Formulation assays further demonstrated that TiO_2_-based protectants can partially preserve viral infectivity under irradiation, underscoring the potential of formulation strategies to enhance field persistence.

Taken together, these findings reveal subtle yet meaningful differences between C168 and Tb that can be attributed to genomic variations identified by deep sequencing. Importantly, C168 displayed significantly higher lethality than Tb, particularly at lower concentrations (1.00 × 10^6^ OBs/mL, *p* < 0.0001), highlighting the potential functional consequences of genetic diversity within natural baculovirus populations [23,31]. Such variation likely reflects genotype competition during transmission and persistence, processes previously recognized as major drivers of baculovirus population structure [32].

Over 450 SNPs were identified, nearly 130 of which were non-synonymous, indicating a wide spectrum of potential protein-level consequences (Table S2). The persistence of such heterogeneity has been attributed to selective pressures in the field and the adaptive value of maintaining multiple genotypes, which may buffer viral populations against stochastic environmental changes and facilitate survival across heterogeneous agroecosystems [33,34,35]. However, the consequences of this diversity are not always straightforward: in vitro cloning often selects variants better adapted to cell culture, which may not reflect the most prevalent or virulent genotypes in the field [36]. Moreover, interactions among co-infecting genotypes can result in synergistic or antagonistic effects, sometimes reducing overall pathogenicity and/or speed of kill compared to individual variants [24,37,38]. These findings emphasize the ecological and applied significance of ChinNPV genetic diversity, particularly for the development of biocontrol products, as variant composition and interactions must be carefully considered in the formulation of sustainable viral insecticides [39]. Future population-level studies including a broader and geographically resolved sampling of ChinNPV field isolates will be required to test whether isolation by distance contributes to the observed phylogenetic relationships suggested in Figure 1B.

Gene variations were distributed across structural, regulatory, and infection-related genes, reinforcing their potential role in shaping phenotypic differences. The *bro-a* gene stood out as highly polymorphic, with 24 SNPs (14 non-synonymous) and a frameshift mutation, consistent with previous reports of *bro* genes as hotspots of plasticity with potential regulatory and structural functions [40,41]. Genes coding for structural proteins also exhibited variability. For instance, *polyhedrin*, whose product is the major constituent of OBs, contained a predicted conservative R > K substitution that may still influence crystal packing and stability [42]. OB morphology is an unreliable taxonomic proxy in Plusiinae: despite tetrahedral OBs reported for RanuNPV, ChinNPV#1, ThorSNPV and a TnSNPV isolate [43,44,45,46], polyhedrin swaps or a single Ile > Leu change at residue 43 in AcMNPV polyhedrin are sufficient to induce tetrahedral crystals in an AcMNPV genetic context [47], while Leu at that position also occurs in Clade II.a viruses that form polyhedral OBs, an outcome best explained by genetic context-dependent, variant-specific effects. On the other hand, genes encoding the capsid-associated protein VP39 and envelope/midgut-entry proteins (FP25K, ODV-E66, PIF-1, PIF-3) showed non-synonymous mutations that could impact capsid assembly, midgut infection efficiency, or virion stability [20,21,48,49,50]. Regulatory and replication-associated genes such as *ie-1, dnapol, dbp, helicase*, and several *lef* genes also carried mutations, which may alter transcriptional activation, replication fidelity, or the timing of late gene expression [50,51,52]. In a previous work analyzing two isolates of ChinNPV, ChinNPV-K and E, of the 394 SNPs detected in the K variant genome, 23 were located in genes directly involved in genome replication (*dna pol*, *helicase*, *alk-exo*) [39]. An additional 43 SNPs were located in genes encoding proteins possibly involved in genome replication, several of which have DNA binding activity (*39 k*/*pp31*, *pcna*, *bro-a*, *bro-b*, *ie-1*, *orf23*) [39]. Authors concluded that the ChinNPV-K variant acts as an unusual generator of variability, introducing adaptive diversity through SNP accumulation and founder effects, in contrast to the stable ChinNPV-E variant.

Certain genes were particularly relevant to isolate-specific traits. The *fgf* gene, which promotes basal lamina remodeling via host metalloproteinases and caspases [53], was present in both isolates but with structural differences: C168 carried an additional valine at position 269, whereas Tb contained a deletion unique among known ChinNPV isolates [23]. This deletion may be associated with the phenotypic divergence observed between C168 and Tb. Although both isolates displayed similar speed of kill (ST_50_ = 6 days) and concentration-dependent developmental disruption, C168 caused a significant reduction in larval growth at lower concentrations compared to Tb (Figure 3), consistent with its higher potency (lower LC_50_). These results do not support a trade-off but rather suggest that genomic variations in key genes such as *fgf* may contribute to differences in virulence intensity and sublethal effects. The *ptp* gene, implicated in host behavior modification and transmission [54], and *egt*, which suppresses molting by blocking ecdysteroid activity [55], were also conserved in both isolates, potentially contributing to the observed one-day prolongation of larval development. Such findings highlight how even small genetic changes in key virulence genes may shift the balance between lethal and sublethal effects, shaping virus–host interactions. The observed short *pif-2* variant, previously reported only in ChinNPV-IA and Chrysodeixis chalcites NPV [18], also raises questions about recombination and gene retention/loss dynamics in ChinNPVs [46].

Phenotypically, both isolates impaired larval growth, pupation, and adult emergence in a concentration-dependent manner. While C168 caused greater reductions in adult emergence at higher concentrations (down to 35.47% at 5 × 10^5^ OBs/mL), Tb exhibited more consistent sublethal effects across all tested concentrations. Virus-treated larvae were significantly smaller, often failed to pupate normally, and surviving adults displayed wing deformities, suggesting cumulative fitness costs that extend beyond lethality (data not shown). Although reproductive parameters such as fecundity and egg hatch were not assessed here, prior work in Plodia interpunctella granulovirus (PlinGV) showed that viral DNA can persist into subsequent generations, being detected in 60–80% of the offspring of survivors [56]. Such persistence mechanisms could apply to ChinNPV, raising the possibility of vertical or transstadial transmission, although our study did not evaluate the presence of viral DNA in pupae or adults, representing a limitation. Nevertheless, the evidence suggests that both isolates influence host life-history traits in ways that may reduce population growth even when mortality is incomplete.

The sensitivity of baculoviruses to UV radiation is a major barrier to large-scale field use. Although OBs provide some physical protection to virions, particularly against desiccation and temperature fluctuations, their shielding capacity against UV radiation is limited, and UV-induced damage (such as pyrimidine dimers and strand breaks) still occurs over time, leading to progressive loss of viral infectivity [11,12,57,58,59]. Here, both ChinNPV isolates were fully inactivated after short UV-C exposures, causing no larval mortality. Previous studies have shown that UV tolerance can arise through natural variation, selective pressure, or genetic engineering, for example, heterogeneous responses in Galleria mellonella NPV [11], selection of AcMNPV variants with altered virulence after repeated UV exposure [60], and the derivation of resistant strains in PlinGV, Cydia pomonella GV, Heliothis NPV, and Helicoverpa armigera NPV [57,58,59,60]. Isolate-specific differences have also been reported for Cryptophlebia leucotreta GV, where the South African isolate (SA) achieved >10^3^-fold increases in tolerance linked to non-synonymous SNPs in *pif-2*, *metalloproteinase*, and *granulin* genes [16]. Many baculoviruses, including ChinNPV, encode class II CPD photolyases that seems to be able to repair UV lesions [17,19,45,46], and expression of a Spodoptera littoralis granulovirus photolyase in Spodoptera littoralis NPV markedly improved UV resistance [21,49]. Notably, despite 431 SNPs between genomes, no variation was found in the *phr* gene of the C168 and Tb isolates, suggesting their UV sensitivity is not tied to photolyase diversity. Formulation assays with C168 showed that kaolin wettable powder offered no protection, whereas the addition of TiO_2_ partially preserved infectivity [21]. Thus, although ChinNPV is intrinsically UV-sensitive, persistence may be improved by combining natural genetic diversity, targeted engineering, and optimized formulations, thereby maintaining efficacy in field conditions and strengthening its role in integrated pest management.

## 5. Conclusions

Our integrative genomic–phenotypic analysis confirms that C168 and Tb are conspecific within *Alphabaculovirus chrincludentis* yet differ in performance in ways that matter for field use, not only in lethality but also in sublethal phenotypes. Despite high genome identity and 431 SNPs (122 nonsynonymous), the *phr* (photolyase) gene was conserved in both isolates. C168 was about 2-fold more potent at low concentrations and both isolates displayed similar ST_95_ (about 6–8 days). However, both viruses exhibited significant developmental costs for *C. includens*, including concentration-dependent reductions in larval growth, pupation, and adult emergence. Notably, sublethal effects were detected at lower concentrations for C168 than for Tb, consistent with its higher potency. Both isolates were completely inactivated by short UV-C exposures, marking UV sensitivity as a primary bottleneck; formulation tests limited to the more lethal C168 showed kaolin was ineffective, while TiO_2_ offered only partial protection. Overall, our findings support the efficacy-driven use of ChinNPV in Brazil, with host specificity and safety as complementary benefits.

## Figures and Tables

**Table 1:** General characteristics of 14 isolates of Chrysodeixis includens nucleopolyhedrovirus (ChinNPV) including genome size, origin and date of collection, annotated ORFs and identity to isolate IE together with the two novel isolates, CNPSo-168 (C168) and Tabatinga (Tb).

Isolates	Size (bb)	Origin/Date *	ORFs	Accession	ID to IE (%) ^§^
ChinNPV-IA	140,808	Guatemala/1972	142	KU669289	97.07
ChinNPV-IB	138,869	Londrina-PR/Jan/2006	141	KU669290	97.72
ChinNPV-IC	140,859	Maringá-PR/Jan/2006	142	KU669291	96.64
ChinNPV-ID	140,787	Iguaraçu-PR/Fev/2006	142	KU669292	96.57
ChinNPV-IE	139,132	Iguaraçu-PR/Fev/2007	141	KJ631622	100.00
ChinNPV-IF	139,181	Dourados-MS/Fev/2007	141	KU669293	99.29
ChinNPV-IG	139,116	Sertanópolis-PR/Jan/2008	141	KU669294	99.3
ChinNPV-MG.A	139,470	MG/Feb-2014	140	MN542939	99.16
ChinNPV-MG.B	139,637	MG/Feb-2014	140	MN542938	98.98
ChinNPV-MT.A	139,113	MT/Jan-2014	140	MN689112	98.98
ChinNPV-MT.B	139,074	MT/Jan-2014	140	MN689113	99.00
ChinNPV-MT.C	138,760	MT/Mar-2014	140	MN689114	98.45
ChinNPV-MT.D	139,046	MT/May-2014	140	MN689115	98.92
ChinNPV-MT.E	139,225	MT/Jun/2014	140	MN689116	98.40
ChinNPV-C168	139,290	Iguaraçu-PR/Jan/2013	154	PX425276	99.44
ChinNPV-Tb	139,065	Tabatinga-DF/?	153	PX425275	98.92

*: When available, the origin includes the city and the month/year of collection. MG: Minas Gerais State; MS: Mato Grosso do Sul State; PR: Paraná State; MT: Mato Grosso State; ^§^: Whole-genome nucleotide identity percentage to ChinNPV-IE.

**Table 2:** Lethal concentrations (LC) and fiducial limits for ChinNPV-C168 and -Tabatinga isolates obtained by logit regression (quasi-binomial model).

Isolate	LC (OB/mL)		Lower	Upper	Slope ± SE	χ^2^ (df; *p*)	Dispersion (φ)
C168	LC_50_ =	5.62 × 10^5^	4.16 × 10^5^	7.58 × 10^5^	2.45 ± 0.30	21.4 (13; 0.064)	1.65
	LC_95_ =	8.90 × 10^6^	4.62 × 10^6^	1.72 × 10^7^			
Tb	LC_50_ =	1.19 × 10^6^	9.49 × 10^5^	1.49 × 10^6^	2.60 ± 0.24	14.2 (13; 0.362)	1.09
	LC_95_ =	1.62 × 10^7^	9.64 × 10^6^	2.73 × 10^7^			

**Table 3:** Effects of ChinNPV isolates CNPSo-168 (C168) and Tabatinga (Tb) on pupation and adult emergence rates of survived *C. includens* exposed to different viral concentrations.

Treatments (OBs/mL)	Survivors (R1/R2/R3)	Pupae (R1/R2/R3)	Adult (R1/R2/R3)	Pupae (%) ^1^	Adult (%) ^1^
**Control**					
0.00 × 10^0^	38/40/40	38/40/40	38/40/40	100.00 ± 0.00	100.00 ± 0.00
**C168**					
1.00 × 10^4^	27/27/28	20/24/27	16/23/25	86.46 ± 11.37	77.91 ± 16.28 *
5.00 × 10^4^	26/24/27	22/19/21	22/19/19	80.51 ± 3.61	78.05 ± 7.18 *
1.00 × 10^5^	22/27/19	17/25/18	17/15/14	88.20 ± 9.52	68.83 ± 11.64 *
5.00 × 10^5^	8/14/5	8/4/3	2/3/3	62.85 ± 35.79 *	35.47 ± 21.31 *
**Tb**					
1.00 × 10^4^	26/26/28	22/22/24	19/21/20	84.98 ± 0.63	75.09 ± 4.98 *
5.00 × 10^4^	29/22/27	22/18/23	21/18/21	80.95 ± 4.72	77.33 ± 4.71 *
1.00 × 10^5^	25/28/25	22/24/25	18/21/21	91.23 ± 7.67	77.00 ± 6.24 *
5.00 × 10^5^	17/12/14	8/6/8	8/3/8	51.40 ± 5.18 *	43.06 ± 16.43 *

^1^: Values represent the mean percentage (± standard deviation) of survivors reaching the pupal and adult stages. Statistical differences relative to the control were assessed using a generalized linear model (GLM, binomial distribution, logit link), followed by pairwise comparisons with estimated marginal means (emmeans) (*p* < 0.001). Asterisks (*) indicate significant differences from the control group. R1/R2/R3: independent biological replicates.

## Data Availability

The original contributions presented in this study are included in the article/supplementary material. Further inquiries can be directed to the corresponding author(s).

## Supplementary Materials

The following supporting information can be downloaded at: https://www.mdpi.com/article/10.3390/v17111503/s1; Table S1. ORF content of the ChinNPV-CNPSo-168 genome (gene name, position, direction and product description); Table S2. ORF content of the ChinNPV-Tabatinga genome (gene name, position, direction and product description); Table S3. Characteristics of single-nucleotide polymorphisms (SNPs) between the Tabatinga and CNPSo168 isolates within ORFs.

## Abbreviations

The following abbreviations are used in this manuscript:

AI	Artificial intelligence
bp	Base pairs
BRM	Microbial Resources Bank (Embrapa/CENARGEN strain code)
C168	ChinNPV-CNPSo-168 isolate
CNPSo	Embrapa Soybean Microbial Collection
ChinNPV	Chrysodeixis includens nucleopolyhedrovirus
*cpd*	Cyclobutane pyrimidine dimer
df	Degrees of freedom
*egt*	Ecdysteroid UDP-glucosyltransferase (viral gene)
EMBRAPA	Brazilian Agricultural Research Corporation
*fgf*	Viral fibroblast growth factor homolog
FP25K	Viral structural protein FP25K
GTR	General Time Reversible (substitution model)
IPM	Integrated pest management
K2P	Kimura two-parameter (distance model)
LC50	Lethal concentration for 50% mortality
LC95	Lethal concentration for 95% mortality
*lef*	Late expression factor genes
MAFFT	Multiple Alignment using Fast Fourier Transform
m.r.c.a.	Most recent common ancestor
NCBI	National Center for Biotechnology Information
OB	Occlusion body
ORF	Open reading frame
*phr*	Photolyase gene
*pif*	Per os infectivity factor (e.g., PIF-1, PIF-3)
*ptp*	Protein tyrosine phosphatase (viral gene)
*rr1*	Ribonucleotide reductase large subunit
SH-like	Shimodaira–Hasegawa-like (branch support measure)
SNP	Single-nucleotide polymorphism
ST50	Median survival time (time to 50% mortality)
Tb	ChinNPV-Tabatinga isolate
UV	Ultraviolet radiation
WP	Wettable powder (formulation)
