# Supplementary material for: Exploring Genomic Variations and Phenotypic Traits of Chrysodeixis includens Nucleopolyhedrovirus Isolates to Improve Soybean Pest Control
Source: Viruses. 2025 Nov 14;17(11):1503. doi: 10.3390/v17111503 (PMC12656735; doi:10.3390/v17111503)
Supplement: Supplementary file 1 [file viruses-17-01503-s001.zip › Table S1.pdf]

Table S1. ORF content of the ChinNPV-CNPSo-168 genome (gene name, position, direction and product description)

ORF	Gene	Direction			Description
1	*polyhedrin*	1	>	741	ORF1, polh, Polyhedrin
2	*ORF1629*	738	<	1937	ORF2, ORF1629, hypothetical protein
3	*pk-1*	1961	>	2779	ORF3, pk1, Protein kinase 1
4	*hoar*	2804	<	4828	ORF4, similar to Tn4, HOAR
5		4807	<	5196	ORF5, hypothetical protein
6		5292	>	5909	ORF6, hypothetical protein
7		6509	>	7594	ORF7, similar to Chch6, hypothetical protein
8	*odv-e66*	7693	>	8808	ORF8, odv-e56/pif-5
9	*me53*	8907	<	10013	ORF9, me53, Major early protein 53
10	*exon0/ie-0*	10309	>	11271	ORF10, exon-0/ie-0, Immediate early protein 0
11	*p49*	11288	>	12715	ORF11, similar to Chch11, P49
12	*odv-e18*	12725	>	12970	ORF12, odv-e18
13	*odv-e27*	13011	>	13892	ORF13, odv-ec27
14		13898	>	14179	ORF14, similar to Chch14, hypothetical protein
15		14230	<	14853	ORF15, similar to Chch15, hypothetical protein
16	*ie-1*	14892	>	17189	ORF16, ie-1, Immediate early protein 1
17	*p74*	17349	>	19325	ORF17, p74/pif-0, Per os infectivity factor 0
18	*p10*	19369	<	19644	ORF18, P10
19	*p26a*	19696	<	20547	ORF19, P26a
20		20708	>	21007	ORF20, similar to Chch20, hypothetical protein
21	*lef-6*	21036	<	21506	ORF21, lef-6, Late expression factor 6
22	*dbp*	21518	<	22501	ORF22, DNA-binding protein
23		22500	>	22667	ORF23, hypothetical protein
24		22600	>	23031	ORF24, similar to Chch23, hypothetical protein
25		23086	>	24315	ORF25, similar to Chch24, hypothetical protein
26		24452	<	25096	ORF26, similar to Tn24, hypothetical protein
27		25071	<	25223	ORF27, hypothetical protein
28		25202	>	25513	ORF28, v-ubi, Viral ubiquitin
29		25431	>	25661	ORF29, similar to ORF25b TnSNPV, hypothetical protein
30	*39k*	25748	<	26725	ORF30, 39K protein (PP31)
31	*lef-11*	26700	<	27167	ORF31, lef-11, Late expression factor 11
32		26993	<	27850	ORF32, similar to Chch30, hypothetical protein
33		27991	<	28518	ORF33, similar to Chch31, hypothetical protein

34	*bro-a*	28787	>	30184	ORF34, bro-a, baculoviral repeat ORF A
35		30276	<	30794	ORF35, hypothetical protein
36	*p47*	30936	<	32129	ORF36, P47
37		32039	>	32218	ORF37, hypothetical protein
38		32329	>	33081	ORF38, similar to Chch34, hypothetical protein
39		33267	<	33950	ORF39, similar to Chch36, hypothetical protein
40	*lef-8*	34046	<	36880	ORF40, lef-8, Late expression factor 8
41	*bjdp*	36904	>	37944	ORF41, bjdp, Baculovirus J domain protein
42	*iap3*	38018	<	38860	ORF42, iap-3, Inhibitor of apoptosis 3
43		39018	>	39197	ORF43, similar to Tn36, hypothetical protein
44		39194	<	39805	ORF44, similar to Chch40, hypothetical protein
45		39867	>	40283	ORF45, similar to Chch41, hypothetical protein
46		40288	<	41403	ORF46, similar to Chch42, hypothetical protein
47		41444	<	41677	ORF47, similar to Chch43, hypothetical protein
48	*lef-10*	41637	>	41864	ORF48, lef-10, Late expression factor 10
49	*vp1054*	41725	>	42726	ORF49, vp1054, Viral protein 1054
50		42850	>	43086	ORF50, similar to Chch46, hypothetical protein
51		42962	>	43360	ORF51, similar to Tn44, hypothetical protein
52		43418	<	43690	ORF52, hypothetical protein
53		43602	>	44114	ORF53, similar to Chch48, hypothetical protein
54		44123	<	44620	ORF54, similar to Chch49, hypothetical protein
55		44642	<	44917	ORF55, similar to Chch50, hypothetical protein
56	*fp/25K*	45188	<	45874	ORF56, fp/25k, Few polyhedra protein
57	*lef-9*	45957	>	47447	ORF57, lef-9, Late expression factor 9
58		47600	<	47809	ORF58, similar to Chch53, hypothetical protein
59		47964	>	48119	ORF59, hypothetical protein
60		48130	>	48288	ORF60, hypothetical protein
61		48619	>	48876	ORF61, similar to Chch56 Tn52, hypothetical protein
62		48883	>	49272	ORF62, similar to Chch57, hypothetical protein
63	*dnapol*	49294	<	52476	ORF63, dnapol, DNA polymerase
64	*desmoplakin*	52475	>	54610	ORF64, Desmoplakin-like protein
65	*lef-3*	54726	<	56129	ORF65, lef-3, Late expression factor 3
66	*pif-6*	56128	>	56523	ORF66, pif-6, Per os infectivity factor 6
67	*iap-2*	56575	>	57468	ORF67, iap-2, Inhibitor of apoptosis 2

68	*p26b*	57514	>	58251	ORF68, similar to Tn59, P26b
69	*v-cath*	58367	<	59401	ORF69, v-cath, Viral cathepsin
70	*chiA*	59515	>	61257	ORF70, chiA, Chitinase
71	*ORF71*	61344	>	62045	ORF71, similar to Tn62, hypothetical protein
72	*pcna*	62064	<	62855	ORF72, pcna, Proliferating cell nuclear antigen
73	*gp37*	62987	>	63805	ORF73, gp37, Glycoprotein 37 (GP37)
74	*phr*	63833	>	65434	ORF74, phr, CPD-photolyase
75	*bro-b*	65510	<	66970	ORF75, bro-b, baculoviral repeat ORF B
76		67117	<	67374	ORF76, similar to Chch71, hypothetical protein
77		67533	>	68087	ORF77, hypothetical protein
78	*he65*	68238	>	68942	ORF78, he65
79	*ctl*	69037	>	69186	ORF79, ctl, Conotoxin-like protein
80		69218	<	69607	ORF80, similar to Ac84, hypothetical protein
81	*vlf-1*	69716	<	70894	ORF81, vlf-1, Very late factor 1
82		70891	<	71265	ORF82, similar to Chch77, hypothetical protein
83	*gp41*	71288	<	72247	ORF83, gp41, Glycoprotein 41
84		72189	<	72905	ORF84, similar to Tn74, hypothetical protein
85		72793	<	73515	ORF85, hypothetical protein
86	*vp91*	73484	>	75967	ORF86, vp91/P95, Viral protein 91
87	*vp39*	76048	<	77055	ORF87, vp39, Viral protein 39 (VP39
88	*lef-4*	77030	>	78445	ORF88, lef-4, Late expression factor 4
89	*p33*	78550	<	79305	ORF89, similar to Chch84, P33 (SOX)
90		79304	>	79786	ORF90, similar to Chch85, hypothetical protein
91	*odv-e25*	79783	>	80445	ORF91, odv-e25
92	*ORF92*	80385	<	80573	ORF92, hypothetical protein
93	*helicase*	80567	<	84199	ORF93, helicase, p143
94	*odv-e28*	84156	>	84674	ORF94, odv-e28/pif-4, Per os infectivity factor 4
95		84708	>	85316	ORF95, similar to Chch89, hypothetical protein
96		85343	>	85573	ORF96, similar to Chch90, hypothetical protein
97	*38k*	85604	<	86560	ORF97, 38K protein
98	*lef-5*	86453	>	87325	ORF98, lef-5, Late expression factor 5
99	*p6.9*	87319	<	87591	ORF99, P6.9
100		87570	>	87725	ORF100, hypothetical protein
101	*p40*	87678	<	88820	ORF101, P40
102	*p12*	88843	<	89208	ORF102, P12
103	*p45*	89195	<	90331	ORF103, P45
104	*p87*	90366	>	92081	ORF104, p87/vp80
105		92078	>	92263	ORF105, hypothetical protein

106	*odv-ec43*	92247	>	93317	ORF106, odv-ec43
107		93360	>	93638	ORF107, similar to Chch100, hypothetical protein
108	*odv-e66*	93681	<	95708	ORF108, odv-e66
109	*p13*	95794	<	96708	ORF109, P13
110		97027	>	97458	ORF110, similar to Chch100, hypothetical protein
111		97508	<	98503	ORF111, similar to Chch104, hypothetical protein
112		98674	<	99066	ORF112, similar to Chch105, hypothetical protein
113		99260	>	100303	ORF113, similar to Chch106, hypothetical protein
114		100385	<	101071	ORF114, similar to Chc107, hypothetical protein
115		101114	<	102697	ORF115, similar to Chch108, hypothetical protein
116		102789	<	103745	ORF116, similar to Chch109, hypothetical protein
117	*pif-3*	103844	<	104485	ORF117, pif-3, Per os infectivity factor 3
118		104572	<	105000	ORF118, similar to Chch111, hypothetical protein
119	*bro-c*	105125	>	105712	ORF119, bro-c, baculovirus repeat ORF C
120	*sod*	105766	>	106221	ORF120, sod, Superoxide dismutase
121		106295	<	106936	ORF121, similar to Chch116, hypothetical protein
122		107093	<	107365	ORF122, similar to Chch117, hypothetical protein
123		107599	<	108111	ORF123, similar to Chch118, hypothetical protein
124	*dut*	108277	<	108762	ORF124, similar to Chch119, dUTPase
125	*calyx*	109016	>	110005	ORF125, calyx/pep, Calyx/PEP
126	*rr2*	110098	<	111054	ORF126, rr2, Ribonucleotide reductase small subunit
127		111175	>	111573	ORF127, similar to Chch123, hypothetical protein
128		111601	>	112725	ORF128, similar to Chch124 Tn116, hypothetical protein
129		112768	<	114057	ORF129, similar to Chch125, hypothetical protein
130		114059	>	114439	ORF130, similar to Chch126, hypothetical protein
131	*alk-exo*	114441	>	115655	ORF131, alk-exo, Alkaline exonuclease
132		115674	<	116417	ORF132, similar to Chch128, hypothetical protein
133	*fgf*	116700	>	117824	ORF133, fgf, Fibroblast growth factor
134	*pif-1*	117921	<	119477	ORF134, pif-1, Per os infectivity factor 1
135		119527	<	120048	ORF135, similar to Chch132, hypothetical protein
136	*gp16*	120124	<	120411	ORF136, gp16, Glycoprotein 16

137	*p24*	120423	<	121163	ORF137, similar to Chch134, P24
138		121291	>	121695	ORF138, similar to Chch135, hypothetical protein
139	*lef-2*	121625	>	122299	ORF139, lef-2, Late expression factor 2
140	*38.7k*	122358	<	123542	ORF140, similar to Chch137, 38.7k protein
141	*lef-1*	123588	<	124283	ORF141, lef-1, Late expression factor 1
142		124272	>	124733	ORF142, similar to Chch139, hypothetical protein
143	*ptp-2*	124735	<	125232	ORF143, ptp2, Phosphotyrosine phosphatase 2
144	*egt*	125392	>	126957	ORF144, egt, Ecdysteroid UDP-glucosyltransferase
145		127160	>	127705	ORF145, similar to Chch142, hypothetical protein
146		127917	<	130694	ORF146, similar to Chch143, hypothetical protein
147		130806	>	130961	ORF147, hypothetical protein
148	*pkip*	131269	>	131772	ORF148, pkip, Protein kinase interacting protein
149	*arif-1*	131847	<	132833	ORF149, arif-1, Actin rearrangement infectivity factor 1
150	*pif-2*	132828	>	133976	ORF150, pif-2, Per os infectivity factor 2
151		134048	>	134248	ORF151, hypothetical protein
152	*f protein*	134374	<	136350	ORF152, f protein
153	*rr1*	136697	<	139042	ORF153, rr1, Ribonucleotide reductase large subunit
154		139060	<	139230	ORF154, hypothetical protein
