# Supplementary material for: Exploring Genomic Variations and Phenotypic Traits of Chrysodeixis includens Nucleopolyhedrovirus Isolates to Improve Soybean Pest Control
Source: Viruses. 2025 Nov 14;17(11):1503. doi: 10.3390/v17111503 (PMC12656735; doi:10.3390/v17111503)
Supplement: Supplementary file 1 [file viruses-17-01503-s001.zip › Table S2.pdf]

Table S2. ORF content of the ChinNPV-Tabatinga genome (gene name, position, direction and product description)

ORF	Gene	Direction			Description
1	*polyhedrin*	1	>	741	ORF1, polh, Polyhedrin
2	*ORF1629*	738	<	1937	ORF2, ORF1629, hypothetical protein
3	*pk-1*	1961	>	2779	ORF3, pk1, Protein kinase 1
4	*hoar*	2804	<	4738	ORF4, similar to Tn4, HOAR
5		4717	<	5043	ORF5, hypothetical protein
6		5311	<	5538	ORF6, hypothetical protein
7		6302	>	7378	ORF7, similar to Chch6, hypothetical protein
8	*odv-e66*	7475	>	8590	ORF8, odv-e56/pif-5
9	*me53*	8689	<	9795	ORF9, me53, Major early protein 53
10	*exon0/ie-0*	10091	>	11053	ORF10, exon-0/ie-0, Immediate early protein 0
11	*p49*	11070	>	12497	ORF11, similar to Chch11, P49
12	*odv-e18*	12507	>	12752	ORF12, odv-e18
13	*odv-e27*	12793	>	13674	ORF13, odv-ec27
14		13680	>	13961	ORF14, similar to Chch14, hypothetical protein
15		14012	<	14635	ORF15, similar to Chch15, hypothetical protein
16	*ie-1*	14674	>	16974	ORF16, ie-1, Immediate early protein 1
17	*p74*	17135	>	19111	ORF17, p74/pif-0, Per os infectivity factor 0
18	*p10*	19155	<	19430	ORF18, P10
19	*p26a*	19482	<	20333	ORF19, P26a
20		20494	>	20793	ORF20, similar to Chch20, hypothetical protein
21	*lef-6*	20822	<	21292	ORF21, lef-6, Late expression factor 6
22	*dbp*	21304	<	22287	ORF22, DNA-binding protein
23		22286	>	22453	ORF23, hypothetical protein
24		22386	>	22817	ORF24, similar to Chch23, hypothetical protein
25		22872	>	24101	ORF25, similar to Chch24, hypothetical protein
26		24238	<	24888	ORF26, similar to Tn24, hypothetical protein
27		24863	<	25015	ORF27, hypothetical protein
28		24994	>	25305	ORF28, v-ubi, Viral ubiquitin
29		25223	>	25453	ORF29, similar to ORF25b TnSNPV, hypothetical protein
30	*39k*	25540	<	26514	ORF30, 39K protein (PP31)
31	*lef-11*	26489	<	26956	ORF31, lef-11, Late expression factor 11
32		26782	<	27639	ORF32, similar to Chch30, hypothetical protein
33		27780	<	28307	ORF33, similar to Chch31, hypothetical protein

34	*bro-a*	28576	>	29943	ORF34, bro-a, baculoviral repeat ORF A
35		30033	<	30551	ORF35, hypothetical protein
36	*p47*	30694	<	31887	ORF36, P47
37		31797	>	31994	ORF37, hypothetical protein
38		32106	>	32861	ORF38, similar to Chch34, hypothetical protein
39		33047	<	33730	ORF39, similar to Chch36, hypothetical protein
40	*lef-8*	33826	<	36660	ORF40, lef-8, Late expression factor 8
41	*bjdp*	36684	>	37724	ORF41, bjdp, Baculovirus J domain protein
42	*iap3*	37798	<	38640	ORF42, iap-3, Inhibitor of apoptosis 3
43		38798	>	38977	ORF43, similar to Tn36, hypothetical protein
44		38974	<	39588	ORF44, similar to Chch40, hypothetical protein
45		39650	>	40066	ORF45, similar to Chch41, hypothetical protein
46		40071	<	41162	ORF46, similar to Chch42, hypothetical protein
47		41203	<	41436	ORF47, similar to Chch43, hypothetical protein
48	*lef-10*	41396	>	41623	ORF48, lef-10, Late expression factor 10
49	*vp1054*	41484	>	42485	ORF49, vp1054, Viral protein 1054
50		42609	>	42845	ORF50, similar to Chch46, hypothetical protein
51		42721	>	43113	ORF51, similar to Tn44, hypothetical protein
52		43171	<	43443	ORF52, hypothetical protein
53		43355	>	43867	ORF53, similar to Chch48, hypothetical protein
54		43876	<	44379	ORF54, similar to Chch49, hypothetical protein
55		44401	<	44676	ORF55, similar to Chch50, hypothetical protein
56	*fp/25K*	44948	<	45634	ORF56, fp/25k, Few polyhedra protein
57	*lef-9*	45717	>	47207	ORF57, lef-9, Late expression factor 9
58		47314	<	47589	ORF58, similar to Chch53, hypothetical protein
59		47744	>	47899	ORF59, hypothetical protein
60		47910	>	48068	ORF60, hypothetical protein
61		48399	>	48656	ORF61, similar to Chch56 Tn52, hypothetical protein
62		48663	>	49052	ORF62, similar to Chch57, hypothetical protein
63	*dnapol*	49074	<	52256	ORF63, dnapol, DNA polymerase
64	*desmoplakin*	52255	>	54390	ORF64, Desmoplakin-like protein
65	*lef-3*	54506	<	55909	ORF65, lef-3, Late expression factor 3
66	*pif-6*	55908	>	56303	ORF66, pif-6, Per os infectivity factor 6
67	*iap-2*	56355	>	57248	ORF67, iap-2, Inhibitor of apoptosis 2

68	*p26b*	57294	>	58031	ORF68, similar to Tn59, P26b
69	*v-cath*	58126	<	59163	ORF69, v-cath, Viral cathepsin
70	*chiA*	59277	>	61019	ORF70, chiA, Chitinase
71	*ORF71*	61106	>	61807	ORF71, similar to Tn62, hypothetical protein
72	*pcna*	61825	<	62616	ORF72, pcna, Proliferating cell nuclear antigen
73	*gp37*	62748	>	63566	ORF73, gp37, Glycoprotein 37 (GP37)
74	*phr*	63594	>	65195	ORF74, phr, CPD-photolyase
75	*bro-b*	65271	<	66731	ORF75, bro-b, baculoviral repeat ORF B
76		66878	<	67135	ORF76, similar to Chch71, hypothetical protein
77		67294	>	67848	ORF77, hypothetical protein
78	*he65*	67999	>	68703	ORF78, he65
79	*ctl*	68803	>	68952	ORF79, ctl, Conotoxin-like protein
80		68984	<	69373	ORF80, similar to Ac84, hypothetical protein
81	*vlf-1*	69482	<	70660	ORF81, vlf-1, Very late factor 1
82		70657	<	71031	ORF82, similar to Chch77, hypothetical protein
83	*gp41*	71054	<	72013	ORF83, gp41, Glycoprotein 41
84		71955	<	72671	ORF84, similar to Tn74, hypothetical protein
85		72559	<	73281	ORF85, hypothetical protein
86	*vp91*	73250	>	75733	ORF86, vp91/P95, Viral protein 91
87	*vp39*	75814	<	76821	ORF87, vp39, Viral protein 39 (VP39
88	*lef-4*	76796	>	78211	ORF88, lef-4, Late expression factor 4
89	*p33*	78317	<	79072	ORF89, similar to Chch84, P33 (SOX)
90		79071	>	79553	ORF90, similar to Chch85, hypothetical protein
91	*odv-e25*	79550	>	80212	ORF91, odv-e25
92	*ORF92*	80152	<	80340	ORF92, hypothetical protein
93	*helicase*	80334	<	83966	ORF93, helicase, p143
94	*odv-e28*	83923	>	84441	ORF94, odv-e28/pif-4, Per os infectivity factor 4
95		84475	>	85083	ORF95, similar to Chch89, hypothetical protein
96		85110	>	85340	ORF96, similar to Chch90, hypothetical protein
97	*38k*	85371	<	86327	ORF97, 38K protein
98	*lef-5*	86220	>	87092	ORF98, lef-5, Late expression factor 5
99	*p6.9*	87086	<	87358	ORF99, P6.9
100		87337	>	87492	ORF100, hypothetical protein
101	*p40*	87445	<	88587	ORF101, P40
102	*p12*	88610	<	88975	ORF102, P12
103	*p45*	88962	<	90098	ORF103, P45
104	*p87*	90133	>	91848	ORF104, p87/vp80
105		91845	>	92030	ORF105, hypothetical protein

106	*odv-ec43*	92014	>	93084	ORF106, odv-ec43
107		93127	>	93405	ORF107, similar to Chch100, hypothetical protein
108	*odv-e66*	93448	<	95475	ORF108, odv-e66
109	*p13*	95561	<	96475	ORF109, P13
110		96794	>	97225	ORF110, similar to Chch100, hypothetical protein
111		97274	<	98323	ORF111, similar to Chch104, hypothetical protein
112		98478	<	98870	ORF112, similar to Chch105, hypothetical protein
113		99060	>	100103	ORF113, similar to Chch106, hypothetical protein
114		100185	<	100871	ORF114, similar to Chc107, hypothetical protein
115		100914	<	102497	ORF115, similar to Chch108, hypothetical protein
116		102592	<	103548	ORF116, similar to Chch109, hypothetical protein
117	*pif-3*	103647	<	104288	ORF117, pif-3, Per os infectivity factor 3
118		104377	<	104805	ORF118, similar to Chch111, hypothetical protein
119	*bro-c*	104930	>	105517	ORF119, bro-c, baculovirus repeat ORF C
120	*sod*	105571	>	106026	ORF120, sod, Superoxide dismutase
121		106100	<	106741	ORF121, similar to Chch116, hypothetical protein
122		106898	<	107170	ORF122, similar to Chch117, hypothetical protein
123		107404	<	107916	ORF123, similar to Chch118, hypothetical protein
124	*dut*	108083	<	108568	ORF124, similar to Chch119, dUTPase
125	*calyx*	108822	>	109811	ORF125, calyx/pep, Calyx/PEP
126	*rr2*	109905	<	110861	ORF126, rr2, Ribonucleotide reductase small subunit
127		110981	>	111388	ORF127, similar to Chch123, hypothetical protein
128		111385	>	112509	ORF128, similar to Chch124 Tn116, hypothetical protein
129		112553	<	113842	ORF129, similar to Chch125, hypothetical protein
130		113844	>	114224	ORF130, similar to Chch126, hypothetical protein
131	*alk-exo*	114226	>	115440	ORF131, alk-exo, Alkaline exonuclease
132		115459	<	116202	ORF132, similar to Chch128, hypothetical protein
133	*fgf*	116485	>	117606	ORF133, fgf, Fibroblast growth factor
134	*pif-1*	117703	<	119259	ORF134, pif-1, Per os infectivity factor 1
135		119309	<	119830	ORF135, similar to Chch132, hypothetical protein
136	*gp16*	119906	<	120193	ORF136, gp16, Glycoprotein 16

137	*p24*	120205	<	120945	ORF137, similar to Chch134, P24
138		121073	>	121477	ORF138, similar to Chch135, hypothetical protein
139	*lef-2*	121407	>	122081	ORF139, lef-2, Late expression factor 2
140	*38.7k*	122136	<	123320	ORF140, similar to Chch137, 38.7k protein
141	*lef-1*	123366	<	124061	ORF141, lef-1, Late expression factor 1
142		124050	>	124511	ORF142, similar to Chch139, hypothetical protein
143	*ptp-2*	124513	<	125010	ORF143, ptp2, Phosphotyrosine phosphatase 2
144	*egt*	125168	>	126733	ORF144, egt, Ecdysteroid UDP-glucosyltransferase
145		126936	>	127481	ORF145, similar to Chch142, hypothetical protein
146		127689	<	130466	ORF146, similar to Chch143, hypothetical protein
147		130578	>	130733	ORF147, hypothetical protein
148	*pkip*	131041	>	131544	ORF148, pkip, Protein kinase interacting protein
149	*arif-1*	131619	<	132605	ORF149, arif-1, Actin rearrangement infectivity factor 1
150	*pif-2*	132600	>	133748	ORF150, pif-2, Per os infectivity factor 2
151		133820	>	134020	ORF151, hypothetical protein
152	*f protein*	134146	<	136122	ORF152, f protein
153	*rr1*	136470	<	138815	ORF153, rr1, Ribonucleotide reductase large subunit
