# Supplementary material for: Exploring Genomic Variations and Phenotypic Traits of Chrysodeixis includens Nucleopolyhedrovirus Isolates to Improve Soybean Pest Control
Source: Viruses. 2025 Nov 14;17(11):1503. doi: 10.3390/v17111503 (PMC12656735; doi:10.3390/v17111503)
Supplement: Supplementary file 1 [file viruses-17-01503-s001.zip › Table S3 (SNPs).pdf]

Table S3. Characteristics of single nucleotide polymorphisms (SNPs) between the Tabatinga and CNPSo168 isolates within ORFs.

Name	SNP Type	Change	Effect	Position
polyhedrin				
G	SNP (transition)		None	474
A	SNP (transition)	R -> K	Substitution	524
T	SNP (transversion)		None	720
ORF1690				
C	SNP (transition)	N -> D	Substitution	949
C	SNP (transition)		None	684
T	SNP (transition)	V -> I	Substitution	91
pk-1				
A	SNP (transition)		None	12
T	SNP (transition)		None	210
T	SNP (transition)		None	315
G	SNP (transition)		None	336
C	SNP (transition)		None	376
A	SNP (transversion)		None	402
A	SNP (transition)		None	447
T	SNP (transition)		None	495
C	SNP (transition)		None	528
T	SNP (transition)		None	577
C	SNP (transition)		None	699
C	SNP (transversion)		None	705
hoar				
A	SNP (transition)	P -> L	Substitution	1,397
T	SNP (transversion)	A -> E	Substitution	1,394
A	SNP (transition)	P -> S	Substitution	1,39
G	SNP (transition)	I -> T	Substitution	1,388
T	SNP (transition)		None	1,188
A	SNP (transition)	P -> S	Substitution	1,174
A	SNP (transition)		None	366
ORF5				
T	SNP (transition)	D -> N	Substitution	244
C	SNP (transition)	K -> R	Substitution	239
T	SNP (transition)		None	237
G	SNP (transversion)	D -> A	Substitution	233
G	SNP (transversion)	G -> A	Substitution	218
T	SNP (transition)	G -> D	Substitution	86
T	SNP (transition)	G -> D	Substitution	74
T	SNP (transition)	G -> D	Substitution	62
ORF6				
C	SNP (transition)	L -> P	Substitution	344
G	SNP (transition)		None	369
C	SNP (transition)	I -> T	Substitution	431
T	SNP (transition)		None	607

ORF7				
A	SNP (transition)	M -> I	Substitution	438
A	SNP (transition)	E -> K	Substitution	718
A	SNP (transversion)		None	912
C	SNP (transversion)	A -> P	Substitution	949
C	SNP (transversion)	A -> P	Substitution	967
A	SNP (transversion)	P -> T	Substitution	997
odv-e66				
G	SNP (transition)	I -> M	Substitution	3
G	SNP (transition)		None	60
G	SNP (transition)		None	141
T	SNP (transition)		None	225
G	SNP (transition)		None	405
A	SNP (transition)		None	498
G	SNP (transversion)		None	513
T	SNP (transition)		None	540
me53				
G	SNP (transversion)		None	1,065
C	SNP (transition)		None	705
A	SNP (transition)		None	303
T	SNP (transition)		None	201
A	SNP (transition)		None	147
T	SNP (transversion)		None	72
C	SNP (transition)	K -> R	Substitution	14
exon0/ie-0				
G	SNP (transversion)	S -> C	Substitution	161
T	SNP (transversion)		None	351
C	SNP (transition)		None	390
A	SNP (transition)	C -> Y	Substitution	431
C	SNP (transition)		None	483
p49				
C	SNP (transition)		None	483
G	SNP (transition)		None	666
G	SNP (transition)		None	723
odv-e27				
T	SNP (transition)		None	73
G	SNP (transition)		None	396
A	SNP (transition)		None	696
T	SNP (transition)		None	708
ORF15				
T	SNP (transversion)	T -> N	Substitution	461
T	SNP (transition)		None	231
ie-1				
G	SNP (transition)	N -> S	Substitution	26
G	SNP (transition)		None	387
T	SNP (transversion)	K -> M	Substitution	857

p74				
T	SNP (transversion)	L -> F	Substitution	261
C	SNP (transversion)	E -> Q	Substitution	355
A	SNP (transversion)	D -> E	Substitution	402
T	SNP (transversion)		None	528
A	SNP (transition)		None	639
C	SNP (transition)		None	669
C	SNP (transition)		None	729
T	SNP (transition)		None	915
T	SNP (transition)		None	1,431
G	SNP (transition)		None	1,572
T	SNP (transition)	T -> I	Substitution	1,607
C	SNP (transition)		None	1,683
C	SNP (transition)		None	1,803
A	SNP (transversion)	M -> K	Substitution	1,844
p26a				
C	SNP (transversion)	F -> L	Substitution	825
T	SNP (transition)		None	741
G	SNP (transition)		None	183
G	SNP (transversion)		None	54
ORF20				
T	SNP (transition)	S -> F	Substitution	59
A	SNP (transversion)		None	78
A	SNP (transition)		None	288
lef-6				
C	SNP (transition)	Q -> R	Substitution	263
C	SNP (transition)	K -> R	Substitution	179
dbp				
A	SNP (transversion)		None	762
C	SNP (transition)		None	729
T	SNP (transition)		None	570
A	SNP (transition)		None	411
C	SNP (transition)		None	366
C	SNP (transition)		None	354
C	SNP (transversion)	T -> S	Substitution	239
A	SNP (transition)		None	180
T	SNP (transition)		None	81
A	SNP (transition)		None	69
ORF24				
A	SNP (transition)	R -> K	Substitution	68
A	SNP (transition)		None	168
ORF25				
G	SNP (transition)		None	51
C	SNP (transition)		None	765
ORF28				
C	SNP (transition)		None	165

39k				
G	SNP (transition)		None	267
lef-11				
A	SNP (transversion)	C -> F	Substitution	23
A	SNP (transversion)		None	465
ORF32				
T	SNP (transversion)	L -> M	Substitution	436
bro-a				
T	SNP (transversion)		None	30
T	SNP (transversion)	K -> N	Substitution	66
C	SNP (transversion)	E -> D	Substitution	72
A	SNP (transition)		None	108
A	SNP (transversion)		None	111
C	SNP (transition)	V -> A	Substitution	188
G	SNP (transition)	E -> G	Substitution	221
C	SNP (transition)	M -> T	Substitution	230
C	SNP (transition)	L -> S	Substitution	233
A	SNP (transition)		None	291
C	SNP (transversion)		None	315
C	SNP (transversion)		None	318
C	SNP (transversion)		None	322
T	SNP (transition)		None	330
G	SNP (transition)		None	339
ORF35				
G	SNP (transition)		None	288
p47				
G	SNP (transition)		None	1,143
A	SNP (transition)		None	1,011
A	SNP (transition)		None	813
G	SNP (transition)	F -> S	Substitution	377
T	SNP (transition)	V -> M	Substitution	373
C	SNP (transition)		None	351
G	SNP (transversion)		None	291
G	SNP (transversion)		None	147
ORF37				
G	SNP (transition)	N -> S	Substitution	116
ORF38				
T	SNP (transversion)		None	369
C	SNP (transition)	F -> L	Substitution	406
A	SNP (transversion)		None	441
G	SNP (transition)		None	618
T	SNP (transition)		None	654
lef-8				
G	SNP (transition)		None	1,932
A	SNP (transition)		None	1,884
A	SNP (transition)	H -> Y	Substitution	1,495

G	SNP (transition)		None	1,491
G	SNP (transition)		None	1,314
G	SNP (transition)		None	642
G	SNP (transition)		None	387
A	SNP (transition)		None	270
A	SNP (transition)		None	204
T	SNP (transition)		None	177
G	SNP (transversion)		None	174
bjdp				
G	SNP (transition)		None	684
A	SNP (transversion)	H -> N	Substitution	1,015
iap3				
A	SNP (transversion)	T -> S	Substitution	673
G	SNP (transition)		None	615
T	SNP (transition)	V -> I	Substitution	574
C	SNP (transition)		None	48
G	SNP (transition)		None	43
ORF44				
T	SNP (transition)	M -> I	Substitution	330
G	SNP (transition)		None	273
A	SNP (transition)		None	240
T	SNP (transition)		None	186
T	SNP (transition)		None	138
T	SNP (transition)	V -> I	Substitution	22
ORF46				
A	SNP (transition)		None	531
vp1054				
C	SNP (transition)		None	177
C	SNP (transition)		None	219
G	SNP (transition)		None	378
T	SNP (transition)		None	786
T	SNP (transition)		None	816
ORF51				
C	SNP (transition)	V -> A	Substitution	77
C	SNP (transition)		None	189
ORF53				
G	SNP (transition)	S -> G	Substitution	109
C	SNP (transversion)	K -> T	Substitution	176
A	SNP (transition)		None	249
C	SNP (transition)		None	291
ORF55				
G	SNP (transition)		None	114
C	SNP (transversion)		None	87
fp/25K				
G	SNP (transition)		None	424
C	SNP (transition)		None	384

lef-9				
A	SNP (transition)		None	273
dnapol				
C	SNP (transversion)	P -> A	Substitution	3,07
T	SNP (transition)	D -> N	Substitution	2,95
G	SNP (transition)		None	1,455
A	SNP (transition)		None	1,083
desmoplakin				
G	SNP (transition)	S -> G	Substitution	727
iap-2				
G	SNP (transition)	K -> E	Substitution	544
A	SNP (transition)	D -> N	Substitution	658
C	SNP (transversion)	C -> S	Substitution	662
p26b				
C	SNP (transversion)		None	501
v-cath				
A	SNP (transition)		None	636
G	SNP (transition)		None	573
C	SNP (transition)	N -> D	Substitution	193
A	SNP (transversion)	Y -> F	Substitution	101
chiA				
T	SNP (transition)		None	234
A	SNP (transition)	R -> K	Substitution	1,433
ORF71				
C	SNP (transition)		None	27
A	SNP (transition)	V -> M	Substitution	364
pcna				
A	SNP (transition)		None	702
T	SNP (transversion)		None	264
A	SNP (transition)		None	252
gp37				
G	SNP (transition)		None	318
bro-b				
C	SNP (transition)		None	1,323
A	SNP (transition)		None	1,248
ORF76				
C	SNP (transversion)	P -> A	Substitution	244
ORF82				
G	SNP (transition)		None	150
gp41				
C	SNP (transition)		None	852
G	SNP (transition)		None	315
vp39				
C	SNP (transition)		None	435
C	SNP (transversion)		None	192

lef-4				
T	SNP (transition)	A -> V	Substitution	569
T	SNP (transition)		None	948
G	SNP (transition)	K -> E	Substitution	1,063
T	SNP (transition)		None	1,083
C	SNP (transition)		None	1,128
C	SNP (transition)		None	1,266
ORF90				
A	SNP (transition)		None	114
odv-e25				
T	SNP (transition)		None	501
G	SNP (transition)		None	552
helicase				
G	SNP (transition)		None	3,534
A	SNP (transversion)	Y -> F	Substitution	3,437
G	SNP (transition)		None	3,33
A	SNP (transition)		None	3,016
G	SNP (transition)	I -> T	Substitution	2,324
T	SNP (transition)		None	2,289
T	SNP (transition)		None	1,965
C	SNP (transition)	N -> S	Substitution	1,874
A	SNP (transition)		None	1,602
A	SNP (transition)		None	1,518
G	SNP (transition)		None	1,507
A	SNP (transition)		None	1,299
T	SNP (transition)		None	1,182
A	SNP (transition)		None	48
ORF96				
A	SNP (transition)	R -> K	Substitution	68
G	SNP (transversion)	D -> E	Substitution	105
lef-5				
C	SNP (transition)		None	423
C	SNP (transition)		None	627
T	SNP (transition)		None	858
p40				
G	SNP (transversion)		None	228
G	SNP (transition)		None	219
G	SNP (transition)		None	210
p12				
A	SNP (transition)		None	120
A	SNP (transition)	T -> I	Substitution	74
p45				
C	SNP (transition)		None	1,104
G	SNP (transition)		None	1,005
A	SNP (transition)		None	993
T	SNP (transition)		None	873

A	SNP (transition)		None	513
T	SNP (transition)		None	495
A	SNP (transition)		None	444
p87				
A	SNP (transversion)	T -> N	Substitution	914
G	SNP (transition)		None	960
A	SNP (transition)	M -> I	Substitution	1,02
G	SNP (transition)	T -> A	Substitution	1,027
T	SNP (transition)		None	1,569
ORF105				
G	SNP (transition)	M -> V	Substitution	70
odv-ec43				
T	SNP (transition)		None	36
C	SNP (transition)		None	409
A	SNP (transition)		None	585
A	SNP (transition)		None	696
C	SNP (transition)		None	771
odv-e66				
A	SNP (transition)		None	843
G	SNP (transition)		None	741
A	SNP (transition)	A -> V	Substitution	680
A	SNP (transition)		None	186
p13				
T	SNP (transition)		None	633
G	SNP (transition)		None	327
A	SNP (transversion)		None	279
G	SNP (transversion)		None	216
ORF111				
C	SNP (transversion)	N -> K	Substitution	990
A	SNP (transversion)	D -> Y	Substitution	913
A	SNP (transition)		None	333
A	SNP (transversion)	K -> I	Substitution	128
C	SNP (transition)		None	108
ORF112				
G	SNP (transition)		None	163
T	SNP (transition)	M -> I	Substitution	63
G	SNP (transition)		None	6
ORF113				
C	SNP (transition)		None	108
T	SNP (transition)		None	120
T	SNP (transition)		None	138
G	SNP (transition)		None	546
C	SNP (transition)		None	606
G	SNP (transition)		None	621
G	SNP (transition)		None	648
T	SNP (transition)		None	798

T	SNP (transition)		None	996
A	SNP (transition)		None	1,035
ORF114				
A	SNP (transition)		None	627
A	SNP (transition)		None	573
C	SNP (transition)		None	555
C	SNP (transversion)		None	507
G	SNP (transition)		None	477
C	SNP (transition)		None	447
G	SNP (transition)		None	102
ORF115				
C	SNP (transition)		None	1,116
C	SNP (transition)		None	819
G	SNP (transition)		None	99
ORF116				
A	SNP (transition)		None	912
A	SNP (transition)		None	894
C	SNP (transition)		None	762
G	SNP (transversion)	Q -> H	Substitution	435
A	SNP (transition)	A -> V	Substitution	392
A	SNP (transition)		None	325
A	SNP (transition)		None	312
T	SNP (transition)		None	288
pif-3				
C	SNP (transversion)		None	588
C	SNP (transition)	K -> E	Substitution	103
G	SNP (transition)	I -> T	Substitution	101
sod				
G	SNP (transversion)		None	372
ORF121				
T	SNP (transition)	S -> N	Substitution	317
T	SNP (transition)	E -> K	Substitution	142
ORF122				
A	SNP (transition)		None	159
A	SNP (transition)	L -> F	Substitution	118
A	SNP (transversion)	K -> N	Substitution	18
ORF123				
C	SNP (transition)	T -> A	Substitution	400
C	SNP (transition)		None	396
T	SNP (transition)		None	306
C	SNP (transition)		None	162
G	SNP (transition)		None	63
dut				
T	SNP (transition)	G -> D	Substitution	56
calyx				
G	SNP (transversion)		None	252

C	SNP (transition)		None	900
A	SNP (transversion)		None	915
A	SNP (transition)		None	975
rr2				
A	SNP (transversion)	E -> D	Substitution	948
C	SNP (transition)		None	429
T	SNP (transition)		None	399
C	SNP (transition)		None	330
ORF127				
C	SNP (transition)		None	178
ORF128				
A	SNP (transversion)		None	129
T	SNP (transition)		None	243
A	SNP (transition)		None	288
A	SNP (transition)	R -> K	Substitution	326
T	SNP (transversion)		None	471
ORF129				
C	SNP (transversion)	H -> Q	Substitution	1,134
T	SNP (transition)	G -> S	Substitution	373
T	SNP (transition)	R -> H	Substitution	329
A	SNP (transition)		None	211
T	SNP (transition)	G -> S	Substitution	154
ORF130				
C	SNP (transversion)	K -> T	Substitution	191
alk-exo				
G	SNP (transversion)	H -> Q	Substitution	30
pif-1				
A	SNP (transition)		None	1,182
A	SNP (transversion)	V -> F	Substitution	1,066
T	SNP (transition)		None	891
A	SNP (transition)		None	111
ORF135				
T	SNP (transversion)		None	489
T	SNP (transition)		None	486
A	SNP (transversion)	Y -> F	Substitution	95
T	SNP (transition)	E -> K	Substitution	88
T	SNP (transition)		None	63
p24				
A	SNP (transition)		None	726
T	SNP (transition)	G -> S	Substitution	532
G	SNP (transition)		None	291
ORF138				
C	SNP (transition)		None	207
lef-2				
A	SNP (transversion)		None	75

G	SNP (transition)		None	273
G	SNP (transversion)	I -> S	Substitution	320
G	SNP (transition)		None	399
C	SNP (transition)		None	441
T	SNP (transition)		None	495
38.7k				
C	SNP (transition)		None	972
T	SNP (transition)		None	672
C	SNP (transition)		None	543
C	SNP (transition)	I -> M	Substitution	528
C	SNP (transition)		None	363
C	SNP (transition)	D -> G	Substitution	194
A	SNP (transition)		None	189
T	SNP (transversion)	L -> I	Substitution	13
lef-1				
T	SNP (transition)		None	591
A	SNP (transversion)		None	438
C	SNP (transition)		None	405
egt				
C	SNP (transition)		None	19
G	SNP (transition)	I -> M	Substitution	33
T	SNP (transition)		None	153
G	SNP (transition)		None	189
C	SNP (transition)		None	228
C	SNP (transition)		None	582
A	SNP (transition)		None	588
A	SNP (transition)		None	684
A	SNP (transition)		None	1,122
T	SNP (transition)		None	1,179
ORF145				
A	SNP (transition)		None	261
T	SNP (transition)		None	459
ORF146				
A	SNP (transition)	P -> L	Substitution	2,459
T	SNP (transition)		None	2,457
A	SNP (transition)		None	2,28
A	SNP (transition)	P -> L	Substitution	1,325
C	SNP (transition)	T -> A	Substitution	1,006
A	SNP (transversion)	Y -> F	Substitution	758
ORF147				
G	SNP (transition)	H -> R	Substitution	101
arif-1				
G	SNP (transition)	M -> T	Substitution	974
G	SNP (transition)	V -> A	Substitution	536
C	SNP (transversion)	A -> G	Substitution	401
G	SNP (transition)		None	291

A	SNP (transition)		None	192
pif-2				
C	SNP (transition)		None	525
C	SNP (transition)		None	657
T	SNP (transition)		None	759
G	SNP (transition)		None	933
G	SNP (transition)	N -> S	Substitution	1,028
f protein				
G	SNP (transition)		None	1,833
A	SNP (transition)		None	1,65
T	SNP (transition)		None	1,44
T	SNP (transition)		None	918
A	SNP (transition)		None	690
T	SNP (transversion)		None	555
A	SNP (transition)		None	442
C	SNP (transition)		None	420
T	SNP (transition)		None	387
A	SNP (transition)		None	369
T	SNP (transition)	S -> N	Substitution	62
rr1				
C	SNP (transition)	N -> D	Substitution	2,284
G	SNP (transition)		None	1,767
A	SNP (transition)		None	1,743
C	SNP (transition)		None	1,47
G	SNP (transition)	L -> S	Substitution	1,382
C	SNP (transition)	I -> M	Substitution	1,203
C	SNP (transition)	T -> A	Substitution	1,189
T	SNP (transition)	V -> I	Substitution	1,165
A	SNP (transition)		None	999
G	SNP (transition)		None	993
A	SNP (transition)		None	762
A	SNP (transition)		None	651
C	SNP (transversion)	D -> E	Substitution	399
